# Efficacy and safety of insulin lispro in geriatric patients with type 2 diabetes: a retrospective analysis of seven randomized controlled clinical trials

**DOI:** 10.1007/s40520-013-0125-7

**Published:** 2013-08-20

**Authors:** Bradley H. Curtis, Tina M. Rees, Kim A. Gaskins, Justo Sierra-Johnson, Rong Liu, Honghua H. Jiang, John H. Holcombe

**Affiliations:** 1 Eli Lilly and Company, Indianapolis, IN USA; 2 Lilly Corporate Center, Indianapolis, IN 46285 USA

**Keywords:** Insulin lispro, Type 2 diabetes, Geriatric, Hypoglycemia

## Abstract

**Background and Aims:**

Glycemic control in geriatric patients with type 2 diabetes (T2DM) remains clinically challenging. The objective of this study was to compare the safety and efficacy of insulin lispro in patients ≥65 years (geriatric) to those <65 years (non-geriatric), using a meta-analysis of randomized controlled clinical trials (RCT).

**Methods:**

This is a retrospective analysis of predefined endpoints from an integrated database of seven RCTs of T2DM patients treated with insulin lispro. The primary efficacy measure tested the non-inferiority of insulin lispro (geriatric vs. non-geriatric; non-inferiority margin 0.4 %) in terms of hemoglobin A_1c_ (HbA_1c_) change from baseline to Month 3 (*N* = 1,525), with change from baseline to Month 6 as a supportive analysis (*N* = 885). Changes in HbA_1c_ from baseline were evaluated with an analysis of covariance model. Secondary measures included incidence and rate of hypoglycemia, and incidence of cardiovascular events.

**Results:**

Mean change in HbA_1c_ from baseline to Month 3 was similar for geriatric (−0.97 %) and non-geriatric patients (−1.05 %); least-square (LS) mean difference (95 % CI) was 0.02 % (−0.11, 0.15 %; *p* = 0.756). Similar results were observed in patients treated up to Month 6; LS mean difference (95 % CI) was 0.07 % (−0.12, 0.26 %; *p* = 0.490). Decrease in HbA_1c_ from baseline to Months 3 and 6 was non-inferior in geriatric compared with non-geriatric patients. There were no significant differences in the incidence and the rate of hypoglycemia, incidence of cardiovascular events, or other serious adverse events including malignancy, post-baseline between the two cohorts.

**Conclusion:**

Key measures of efficacy and safety in geriatric patients with T2DM were not significantly different from non-geriatric patients when utilizing insulin lispro. Insulin lispro may be considered a safe and efficacious therapeutic option for the management of T2DM in geriatric patients.

## Introduction

The burden of type 2 diabetes on geriatric patients is an important public health issue. According to the American Diabetes Association (ADA), it was estimated that approximately 26.9 % (~10.9 million) of US residents aged 65 years or older had diabetes, of which ~390,000 were newly diagnosed [1].

While ADA guidelines do not have a specific target recommendation for geriatric patients and contemplate individualization of hemoglobin (Hb) A_1c_ (HbA_1c_) goals for many non-pregnant adults with diabetes [2], American Geriatric Society (AGS) guidelines suggest a target of 8.0 % in frail geriatric patients with diabetes [3]. The principles of blood glucose control are essentially the same in geriatric patients compared to middle-aged adult patients; however, achievement of glycemic control is often complicated by many factors in geriatric patients. Age-related decline in physical and cognitive functions, difficulty in achieving dietary and exercise goals, presence of multiple comorbidities, polypharmacy and increased risk for adverse events are some of the factors noted [4].

This meta-analysis was undertaken using data from seven randomized controlled trials (RCTs) of patients with type 2 diabetes treated with insulin lispro to test the hypothesis that the use of insulin lispro in patients ≥65 years of age is as safe and efficacious to use as it is in patients <65 years of age, as measured by change in HbA_1c_ and rates of hypoglycemia.

## Methods

### Selection criteria and characteristics of clinical studies

An integrated database of RCTs was created using pre-specified criteria from a pool of 18 RCTs using insulin lispro conducted by Eli Lilly and Company. Studies were included only if they met the following inclusion criteria: (1) studies were global in nature and had patient-level data available for inclusion in the analysis; (2) the study population consisted of patients with type 2 diabetes; (3) there was an insulin lispro treatment arm(s) with a treatment period of at least 3 months duration; and (4) HbA_1c_ was measured at baseline (before the study drug was administered) and at least once post-baseline (after the study drug was administered).

The two age cohorts were defined based on patient ages at randomization; the geriatric cohort included patients ≥65 years of age and the non-geriatric cohort included patients <65 years of age. Non-inferiority of geriatric cohort to non-geriatric cohort in terms of HbA_1c_ change from baseline to the endpoint (3 and 6 months of treatment) was considered confirmed with a non-inferiority margin of 0.4 %. This was established if the lower limit of the 95 % confidence interval (CI) of the difference in HbA_1c_ change between the two age cohorts (non-geriatric cohort minus geriatric cohort) was >−0.4 %, based on data for up to 3 and 6 months of treatment. This non-inferiority margin is similar to that used in other treat-to-target studies [5–7]. Secondary objectives included an assessment of the overall incidence and rates of hypoglycemia (all patient-reported events) and cardiovascular event incidence in an HbA_1c_-matched cohort, based on data for up to 3 and 6 months of treatment. See the “Results” section for detailed definitions of hypoglycemia used in studies included in the meta-analysis.

### Statistical methodology

Statistical analyses of the primary and secondary endpoints were performed in accordance with a pre-specified statistical analysis plan based on a modified intent-to-treat (ITT) population with patients who had at least one dose of study insulin and age information available. The last observation carried forward (LOCF) method was used to replace missing values. The primary analyses were performed on the ITT population combining all seven studies with ≥3 months of insulin lispro treatment. HbA_1c_ change from baseline was evaluated with an analysis of covariance (ANCOVA) model that contained baseline HbA_1c_ as a covariate and age cohort (geriatric vs. non-geriatric), study, country (grouped by region: North America, Asia, Africa, and Europe) and basal insulin, use (yes or no) as factors. A study factor was included in all analysis models to adjust for imbalances noted at baseline. Results from the above analyses are presented under headings of “All Studies Combined”. The supportive analyses were performed on the ITT population combining four studies with ≥6 months of insulin lispro treatment. Results from supportive analyses are presented under headings of “Long-term Studies Combined”. Sensitivity analyses that excluded patients with extreme HbA_1c_ values (HbA_1c_ <4.5 %, or HbA_1c_ >15 %) were conducted at baseline, Month 3, and/or at Month 6. These sensitivity analyses showed similar results to that of the whole population and accordingly these patients were included in the full analysis. The rates of all patient-reported hypoglycemia (episodes/patient/30 days) were compared between the age cohorts with a non-parametric ranked analysis of variance (ANOVA) model that contained age cohort, study, and country as factors. In addition to the ranked ANOVA model, a negative binomial model was used as a supportive model for analyzing the number of episodes and was performed for each analysis with the same factors as in the ranked ANOVA model. The incidence of hypoglycemia was analyzed with logistic regression models containing age cohort, study, and country as factors. Since renal function can influence the incidence of hypoglycemia, an analysis was performed including data from four studies that had plasma creatinine data available and patients with impaired renal function (defined as glomerular filtration rate (GFR) <60 mL/min/1.73 m^2^). Insulin dose was analyzed with an ANOVA model that contained age cohort, study, and country. No adjustments for multiplicity were performed. A two-sided *p* value of <0.05 was considered statistically significant.

Cardiovascular events were defined as cardiac disorders at the system–organ–class term level for the purposes of comparison of cardiovascular events across studies. The Cochran–Mantel–Haenszel test was used to evaluate the difference in the overall cardiovascular event rates between the two cohorts after stratifying the data for baseline HbA_1c_ cohort, study, and anti-hyperglycemia concomitant therapy use (yes or no).

## Results

**Fig. 1:**
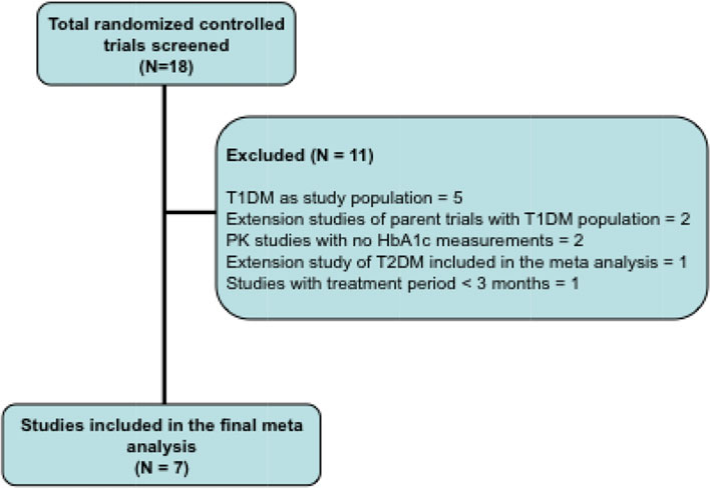
Flow diagram of the study selection process. *HbA*_*1c*_ hemoglobin A_1c_, *PK* pharmacokinetic, *T1DM* type 1 diabetes, *T2DM* type 2 diabetes

Of the 18 studies reviewed, 11 studies did not meet inclusion criteria and were not included in the meta-analysis (Fig. 1). Of these 11 studies, five consisted of patients with type 1 diabetes as the study population, two were extension studies of RCTs with type 1 diabetes population, one was an extension of an included RCT such that the patients were already included in the meta-analysis, one had a treatment period of only 2 months, and two were pharmacokinetic studies with no HbA_1c_ measurements. Therefore, a total of seven RCTs were identified to meet all inclusion criteria and data from patients who were treated with insulin lispro in these trials were included in the final analysis.

Table 1 shows the characteristics of the seven studies selected. Of these studies, three were randomized, parallel group, open-label, active-controlled studies with insulin lispro as one of two treatment arms with a treatment period of 12 months [8, 9]. Both men and women with type 2 diabetes between the ages of 35 and 70 were enrolled in the first two studies. Prior to enrollment, all patients used commercially available human insulin for at least 2 months and had achieved optimum compliance with their diabetic diet and insulin therapy [8]. However, for the third study, only patients with type 2 diabetes who had either not received insulin or had received insulin for <2 months prior to giving their informed consent were allowed to enroll in the study and the study population involved a wider age range (25–82 years) [9].

**Table 1:** Characteristics of clinical studies

Study citation	Primary objective	Study design	Test product and dosage regimen^a^	No. of pts^b^ (geriatric/non-geriatric)^c^	Duration of treatment
Efficacy and safety					
[8]	To compare insulin lispro and regular human insulin with respect to postprandial glucose excursions, frequency of hypoglycemic episodes in relation to glycemic control, metabolic control, and safety in patients with type 2 diabetes	Randomized, parallel, open-label, active controlled	Solution, 100 U/mL (with doses adjusted to meet individual metabolic needs), prior to any meal	157 (9/63)	12 months
[8]	To compare insulin lispro and regular human insulin with respect to postprandial glucose excursions, frequency of hypoglycemic episodes in relation to glycemic control, metabolic control, safety, and immunological response in patients with type 2 diabetes	Randomized, parallel, open-label, active controlled	Solution, 100 U/mL (with doses adjusted to meet individual metabolic needs), prior to any meal	160 (16/57)	12 months
[9]	To compare insulin lispro with regular human insulin with respect to postprandial glucose excursions, incidence of hypoglycemia, and hypoglycemic rate in relation to glycemic control, metabolic control, and safety in patients who were new to insulin therapy for type 2 diabetes	Randomized, parallel, open-label, active controlled	Solution, 100 U/mL (with doses adjusted to achieve pre-defined glycemic control goals), prior to any meal	377 (33/149)	12 months
[10]	To compare insulin lispro and regular human insulin with respect to postprandial glucose excursions, frequency of hypoglycemic episodes in relation to glucose control, metabolic control, and safety in patients with type 2 diabetes	Randomized, 2-period crossover, open-label, active controlled	Solution, 100 U/mL (with doses adjusted to meet individual metabolic needs), prior to any meal	777 (87/266)	6 months (3 months for patients included in this analysis)
[11]	To identify the insulin treatment strategy with the highest success rate regarding use of insulin lispro in patients with type 2 diabetes after oral agent failure. The strategies included	Randomized, open-label, 2-period, active control	Solution, 100 U/mL (with doses adjusted to meet predefined glucose target), prior to any meal	423 (88/184)	4 months
	(1) Prandial insulin treatment using insulin lispro with NPH insulin at bedtime				
	(2) Prandial insulin treatment using insulin lispro in combination with a sulfonylurea; and				
	(3) Insulin treatment with NPH insulin at bedtime in combination with a sulfonylurea				
Safety					
[12]	To demonstrate a difference between	Randomized, open-label, 2-arm, parallel, active controlled	Solution, 100 U/mL (with doses dependent upon the individual requirements), prior to any meal	1,227 (202/356)	18-month initial treatment followed by up to 5.5 years of follow-up; potential total duration 7 years was planned. Study was stopped for futility, actual mean treatment duration was 963 days
	(1) a treatment strategy that targeted postprandial glycemia (administration of insulin lispro; referred to as “postprandial” group), and				
	(2) a treatment strategy that targeted fasting/interprandial glycemia (administration of basal insulin NPH or insulin glargine] or biphasic intermediate-acting insulin [human insulin 30/70], if pre-cardiac care unit admission therapy was such; hereafter referred to as “fasting” group), while aiming to achieve and maintain Hb A_1c_ <7.0 % in both groups in the time until the occurrence of the first event from the combined study outcomes in patients with type 2 diabetes and acute myocardial infarction				
[13]	To characterize acute postprandial markers after a test meal in patients with type 2 diabetes treated with premeal insulin lispro plus bedtime NPH or twice-daily NPH. Postprandial markers evaluated include D-glucitol, high-sensitivity C-reactive protein, nitrotyrosine, Factor VII, prothrombin fragments 1 and 2, total cholesterol, LDL-C, LDL size and oxidation, HDL-C, LDL-C/HDL-C ratio, triglycerides, free fatty acids, and chylomicron remnants	Randomized, open-label, 2-period, crossover, active controlled	Solution, 100 U/mL (with doses adjusted to meet predefined glucose target), prior to any meal	30 (6/9)	6 months (3 months for patients included in this analysis)

*BMI* body mass index, *HDL*-*C* high density lipoprotein cholesterol, *LDL*-*C* low density lipoprotein cholesterol, *NPH* human insulin isophane suspension

^a^All doses were administered subcutaneously in abdomen

^b^Total number of patients enrolled in each study based on the clinical study reports

^c^Number of patients in each age group included in the analysis. Note that patients who were enrolled, but did not receive insulin lispro or were missing data on age, were not included in the analysis

The fourth study was a randomized, two-period crossover, open-label, active-controlled study with two treatment periods of 3 months each and involved patients with type 2 diabetes who had been receiving human insulin for at least 2 months prior to enrolling in this study [10]. At Visit 1 of this study, patients were placed on Humulin R for pre-meal insulin therapy and either Humulin N (NPH) or Humulin U for basal insulin therapy for a 2- to 4-week lead-in period [10]. The fifth study was not a cross-over design but had two treatment periods (period 1 and 2) of 2 months each [11]. This randomized, open-label study compared insulin lispro, sulfonylurea, and NPH insulin in patients with type 2 diabetes who required treatment with insulin after failure of an oral agent therapy. Data from insulin lispro + NPH and insulin lispro + sulfonylurea treatment groups from both treatment periods were included in the meta-analysis.

The sixth study was a randomized, open-label, two-arm parallel study where each patient underwent a lead-in period prior to randomization. This study consisted of an initial 18-month treatment period followed by an extended follow-up phase of up to 5 1/2 years, for a total potential duration of treatment of up to 7 years [12]. Patients with type 2 diabetes and acute myocardial infarction were randomized to one of the two treatment strategies: one treatment strategy that targeted postprandial glycemia (administration of insulin lispro) and another that targeted fasting/interprandial glycemia (administration of basal insulin or biphasic intermediate-acting insulin). Only data from patients randomized to the postprandial group were used in the meta-analysis.

Similar to the fourth study, the seventh study was a multicenter, randomized, open-label, two-period crossover study consisting of an 8-week lead-in treatment with NPH twice daily (BID). Patients were then randomized to receive either pre-meal insulin lispro + bedtime NPH or NPH BID for 3 months each [13]. Data from patients receiving insulin lispro + NPH during period 1 were used in the current analysis. The various definitions of hypoglycemia used in the trials included in the meta-analysis are presented in Table 2.

**Table 2:** Definition of hypoglycemic episodes according to individual studies

Category	Definitions
Hypoglycemic episodes	Any time a patient felt that he or she was experiencing a sign or symptom that he or she associated with hypoglycemia, or a blood glucose measurement of <2.0 mmol/L (36 mg/dL). No definition of severe or serious hypoglycemia was included in the protocol, but characteristics of each episode were collected [8]
	A patient experiencing signs/symptoms of hypoglycemia and/or accompanied by a fingerstick glucose <63 mg/dL (3.5 mmol/l) even if it is not associated with signs, symptoms, or treatment [9, 10, 12, 13]
	A patient experiencing signs/symptoms of hypoglycemia and/or accompanied by a fingerstick glucose <54 mg/dL (3.0 mmol/l) [11]
Severe/serious hypoglycemia	Severe hypoglycemia was defined when assistance from another individual is required because it is disabling, i.e., any episode where the patient is in a coma or requires a glucagon or intravenous glucose injection [13]
	Severe hypoglycemia was defined as fingerstick glucose <50 mg/dL (2.8 mmol/l) [12]
	Serious hypoglycemia was defined when the episode resulted in coma or required treatment with a glucose or glucagon injection [11]

### Patient disposition and baseline characteristics

A total of 1,525 patients from the “all-studies combined” dataset were included in the analysis, of which 885 patients received treatment for up to 6 months (“long-term studies combined” dataset). In the “all-studies combined” analysis, there were 1,084 (71 %) non-geriatric patients and 441 (29 %) geriatric patients. Approximately 94.3 % of non-geriatric patients (1,022/1,084) and 91.6 % of geriatric patients (404/441) completed at least 3 months of insulin therapy. There were no statistically significant differences in the reasons for discontinuation of a study between the age cohorts, except for the reason of death (geriatric: 9 patients, 2.0 %; non-geriatric: 6 patients, 0.6 %; *p* = 0.017). For the “long-term studies combined”, a significantly lower proportion of geriatric patients (84.6 %) completed 6 months of their clinical study compared with non-geriatric patients (90.7 %; *p* = 0.010). Statistical differences between age cohorts were noted for “personal conflict” or “other patient decision” as reasons for discontinuation (data not shown).

Table 3 presents the baseline characteristics of all patients included in each analysis of “all-studies combined” and “long-term studies combined”. The majority of the sample were males, but with an overall smaller proportion of males in the geriatric cohort compared with the non-geriatric cohort. A greater proportion of the geriatric cohort was Caucasian compared with the non-geriatric cohort. The mean duration of diabetes ranged from 8.5 to 12 years and was significantly greater for geriatric patients. At baseline, HbA_1c_ was significantly lower for the geriatric cohort. Further, a smaller proportion of patients in the geriatric cohort reported at least one previous episode of hypoglycemia at the baseline visit. In addition, there was a significantly lower mean baseline rate of hypoglycemia episodes in the geriatric cohort compared with the non-geriatric cohort. Mean BMI was similar for both age cohorts.

**Table 3:** Summary of patient characteristics at baseline

Category	All studies combined (*N* = 1,525)		*p* value	Long-term studies combined (*n* = 885)		*p* value
	Non-geriatric (*n* = 1,084)	Geriatric (*n* = 441)		Non-geriatric (*n* = 625)	Geriatric (*n* = 260)	
Age, mean years (SD)	54.19 (7.32)	70.49 (4.11)	<0.001	54.23 (7.22)	70.85 (4.18)	<0.001
Males, *n* (%)	649 (59.9)	230 (52.2)	0.006	401 (64.2)	132 (50.8)	<0.001
Race/regional origin (%)^a^						
Caucasian	82.6	93.4		78.7	93.5	
Western Asian	7.1	1.8		10.9	2.7	
African descent	4.3	2.3	<0.001	4.8	1.9	<0.001
Hispanic	3.0	0.7		3.0	0.8	
East/Southeast Asian	0.5	0.7		0.3	0	
Other	2.5	1.1		2.2	1.2	
Body weight (kg)						
Mean (SD)	82.43 (16)	78.02 (14.10)	<0.001	82.1 (15.29)	78.05 (13.18)	<0.001
Body mass index (BMI)						
Mean (SD)	28.77 (4.76)	28.41 (4.32)	0.173	28.64 (4.56)	28.56 (4.27)	0.814
Duration of diabetes (years)						
Mean (SD)	9.30 (6.89)	12.33 (8.11)	<0.001	8.53 (6.76)	11.70 (8.24)	<0.001
Baseline HbA_1c_ (%)						
Mean (SD)	9.06 (1.68)	8.77 (1.65)	0.002	8.83 (1.62)	8.40 (1.46)	<0.001
Baseline incidences of hypoglycemia (%)						
None	70.5	79.9	<0.001	80.0	87.7	0.007
At least 1 in pre-study assessment	29.5	20.1		20.0	12.3	
Baseline rate of hypoglycemia (episodes per 30 days, mean (SD))						
Overall	1.66 (3.89)	1.10 (3.30)	0.006	1.03 (0.00)	0.64 (0.00)	0.050

Means were analyzed using a 2-sample *t* test for age, body weight, BMI, duration of diabetes, and baseline HbA_1c_ data

Frequencies are analyzed using Fisher’s exact test for gender, race, and baseline incidences of hypoglycemia data

*p* values were calculated using a ranked ANOVA model to analyze the rate of hypoglycemic episodes per 30 days with age group being the covariate

^a^Significantly different between the two age cohorts

### Efficacy

The primary efficacy endpoint of non-inferiority of insulin lispro treatment in the geriatric cohort as compared to the non-geriatric cohort was achieved for both the “all studies combined” and the “long-term studies combined” analyses (Table 4). The mean change in HbA_1c_ from baseline to Month 3 was similar for patients in the geriatric cohort (−0.97 %) and non-geriatric patients (−1.05 %), with an LS mean difference (95 % CI) of 0.02 % (−0.11, 0.15 %; *p* = 0.756). Since the lower limit of the 95 % CI was greater than −0.4 %, non-inferiority was demonstrated. Basal insulin use was not a significant factor and similar treatment comparison results were observed when patients with HbA_1c_ values defined as outliers (<4.5 % or >15 %) were excluded.

**Table 4:** Changes in HbA_1c_ levels from baseline in type-2 diabetes patients treated with insulin lispro

Age group	All studies combined (*N* = 963)^a^				Long-term studies combined (*n* = 377)^a^			
	Baseline	Month 3 (LOCF)	Change from baseline	LS mean	Baseline	Month 6 (LOCF)	Change from baseline	LS mean
Non-geriatric	9.07 (1.69)	8.03 (1.38)	−1.05 (1.49)	−0.96 (0.10)	8.85 (1.64)	7.68 (1.34)	−1.16 (1.69)	−1.13 (0.12)
Geriatric	8.82 (1.66)	7.85 (1.23)	−0.97 (1.29)	−0.94 (0.08)	8.44 (1.49)	7.48 (1.17)	−0.96 (1.38)	−1.20 (0.15)
Age group comparison^b^	LS mean difference (95 % CI)			0.02 (−0.11, 0.15)	LS mean difference (95 % CI)			0.07 (−0.12, 0.26)
	LS mean difference (*p* value)			0.756	LS mean difference (*p* value)			0.490

*LOCF* last observation carried forward, *n* number of patients included in the analysis

^a^Patients who had only baseline values or those who did not have any baseline values were not included in this analysis. Data are shown as mean (SD) unless otherwise noted

^b^The ANCOVA model included age cohort, basal insulin use, baseline HbA_1c_, country cohort, and study. Age cohort by baseline HbA_1c_ and age cohort by study were not included in the model as these were not statistically significant factors

The mean change in HbA_1c_ from baseline to Month 6 was similar between the age cohorts (geriatric: −0.96 %; non-geriatric: −1.16 %), with an LS mean difference (95 % CI) of 0.07 % (−0.12, 0.26 %; *p* = 0.490). The mean daily insulin lispro dose was similar for the two age cohorts both at Month 3 (geriatric: 0.39 unit/kg; non-geriatric: 0.38 unit/kg, *p* = 0.648) and Month 6 (geriatric: 0.45 unit/kg; non-geriatric: 0.42 unit/kg, *p* = 0.676).

**Fig. 2:**
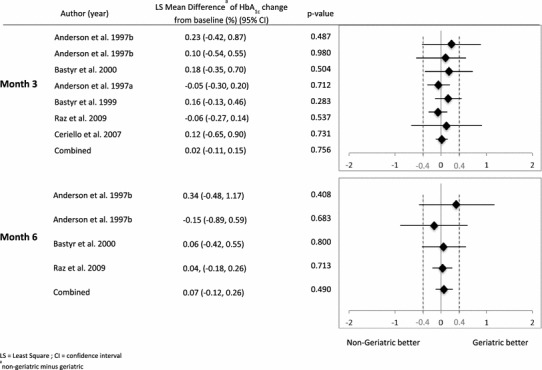
LS mean difference of the change in HbA_1c_ from meta-analysis by study (non-geriatric minus geriatric)

Figure 2 shows the LS mean differences of HbA_1c_ level from baseline to Month 3 (upper panel) and Month 6 (lower panel). There were no significant differences between the two age cohorts across the individual studies included in the meta analyses.

### Hypoglycemia

**Fig. 3:**
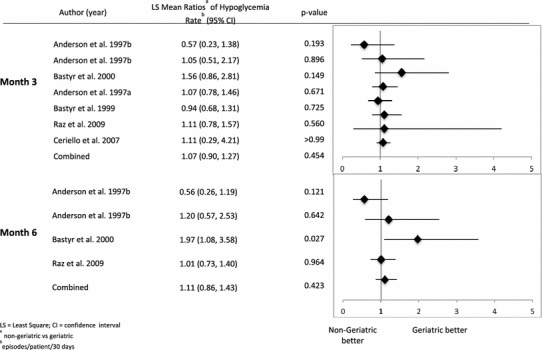
LS mean ratios of hypoglycemia rates from meta-analysis by study (non-geriatric vs. geriatric)

No statistically significant differences were noted in the incidence of hypoglycemic episodes up to Months 3 and 6 between the two age cohorts (Table 5). The mean rate of hypoglycemia was similar for geriatric as well as non-geriatric patients (Month 3: 1.21 vs. 1.52 per 30 days [*p* = 0.276]; Month 6: 0.71 vs. 1.01 per 30 days [*p* = 0.234]). Analysis of the incidence of all patient-reported hypoglycemic episodes as well as the rate of hypoglycemia after adjusting for renal function also showed no statistically significant differences between age cohorts for “all-studies combined” and “long-term studies combined.” The overall incidence of severe hypoglycemia in 2 of the 7 studies that contained the definition was found to be <5 % in both geriatric and non-geriatric cohorts; however, the mean rate of severe hypoglycemia was significantly greater in geriatric compared to non-geriatric cohort up to Month 3 (0.02 and 0.00 per 30 days, respectively; *p* = 0.009) and Month 6 (0.01 and 0.02 per 30 days, respectively; *p* = 0.007 [data not shown in Table]). Figure 3 shows that there is no significant heterogeneity (based on age by study interaction) in relation to the LS mean ratio of hypoglycemic rates observed between geriatric and non-geriatric cohorts.

**Table 5:** Incidence and rate of hypoglycemic episodes

	All studies combined (up to month 3)		*p* value	Long-term studies combined (up to month 6)		*p* value
	Non-geriatric (*n* = 1,084)	Geriatric (*n* = 441)		Non-geriatric (*n* = 625)	Geriatric (*n* = 260)	
Overall incidence of hypoglycemic episodes, *n* (%)	659 (60.79)	241 (54.65)	0.828	368 (58.88)	131 (50.38)	0.941
Overall rate of hypoglycemic episodes per 30 days, mean (SD)	1.52 (2.81)	1.21 (2.47)	0.276	1.01 (1.99)	0.71 (1.67)	0.234

### Cardiovascular safety

The relatively small number of overall cardiac events (defined as cardiac disorders at the system–organ–class term level) in the studies included in this meta-analysis, as well as the short duration of the studies, precluded definitive conclusions regarding the cardiovascular safety profile of insulin lispro in geriatric versus non-geriatric patients. Analysis of cardiovascular events by pre-existing conditions at baseline for Months 3 and 6, based on Fisher’s exact test, showed that a significantly greater proportion of geriatric patients had a previous cardiovascular event compared with non-geriatric patients (Month 3: 43.1 vs. 25.4 %, respectively [*p* < 0.001]; Month 6: 52.7 vs. 31.8 %, respectively [*p* < 0.001]). However, no statistically significant differences were noted between the geriatric and non-geriatric cohorts in the proportion of patients who developed a cardiovascular event after baseline (Month 3: 7.3 vs. 5.4 %, respectively [*p* = 0.152]; Month 6: 14.2 vs. 11.2 %, respectively [*p* = 0.214]) or in the percentage of patients with cardiovascular events at baseline having increased severity of that event during the study (Month 3: 0.5 vs. 0.6 %, respectively [*p* > 0.999]; Month 6: 0 vs. 1.0 %, respectively [*p* = 0.188]). Analysis of cardiovascular events at preferred term level up to Months 3 and 6 by the following strata: anti-hyperglycemia concomitant therapy use (yes/no); baseline HbA_1c_ cohort (<7, 7–8, 8–9, 9–10, >10 %); or study, showed no statistical differences between the two age cohorts based on Cochran–Mantel–Haenszel test.

### Serious adverse events

Overall, a similar percentage of patients experienced at least one serious adverse event (SAE) across the two age cohorts at month 3 (geriatric: 34 patients, 7.7 %; non-geriatric: 60 patients, 5.5 %; *p* = ns) and month 6 (geriatric: 27 patients, 10.4 %; non-geriatric: 51 patients, 8.2 %; *p* = ns). Infection and infestations was the only classification that reached statistical significance in the geriatric cohort compared with non-geriatric at month 3 (geriatric: 6 patients, 1.4 %; non-geriatric: 2 patients, 0.2 %; *p* = 0.009) and at month 6 (geriatric: 6 patients, 2.3 %; non-geriatric: 3 patients, 0.5 %; *p* = 0.022). There was no difference in the rates of malignancy/neoplasms between the two cohorts at both Months 3 and 6.

### Other safety analyses

At Month 3, the most frequently reported (≥2 %) treatment emergent adverse events (TEAEs) for geriatric patients included (in order of decreasing frequency): headache, nasopharyngitis, peripheral odema, arthralgia, cough, and dyspnoea. A significantly lower proportion of geriatric patients had at least one TEAE compared with non-geriatric patients at Month 3 (geriatric: 212 patients, 48.1 %; non-geriatric: 646 patients, 59.6 %;  p < 0.001) and Month 6 (geriatric: 141 patients, 54.2 %; non-geriatric: 392 patients, 62.7 %; *p* = 0.020). In the 3-month analysis, TEAEs occurring significantly more frequently in geriatric patients as compared to non-geriatric patients included arteriogram coronary (geriatric: 10 patients, 3.8 %; non-geriatric: 9 patients, 1.4 %; *p* = 0.038) and hypoglycemia (geriatric: 4 patients, 1.5 %; non-geriatric: 1 patient, 0.2 %; *p* = 0.028). In addition, diabetic retinopathy was reported more frequently in geriatric patients compared to non-geriatric patients (geriatric: 7 patients, 1.6 %; non-geriatric: 5 patients, 0.5 %; *p* = 0.048).

At Month 6, the most frequently reported (≥2 %) TEAEs for geriatric patients were arteriogram coronary, headache, nasopharyngitis, diabetic retinopathy, anemia, bronchitis, dizziness, and rhinitis. TEAEs occurring significantly more frequently in geriatric patients as compared to non-geriatric patients at Month 6 included arteriogram coronary (geriatric: 10 patients, 3.8 %; non-geriatric: 9 patients, 1.4 %; *p* = 0.038) and hypoglycemia (geriatric: 4 patients, 1.5 %; non-geriatric: 1 patient, 0.2 %; *p* = 0.028).

## Discussion

Insulin therapy remains underutilized in geriatric populations despite the fact that many geriatric patients with a history of type 2 diabetes could benefit from the use of insulin to achieve improved glycemic control [14]. This meta-analysis of seven randomized controlled clinical trial data showed non-inferiority in the efficacy of insulin lispro for geriatric patients compared with non-geriatric patients. Despite the heterogenic nature of the seven RCTs analyzed, the mean changes in HbA_1c_ from baseline to Months 3 and 6 were similar for geriatric and non-geriatric patients treated with insulin lispro, and the decrease in HbA_1c_ was associated with the same dose of insulin lispro across both age cohorts.

To date, clinical evidence on achieving maximal glycemic control with minimal adverse effects in geriatric subjects is scarce [15, 16]. Published guidelines for managing diabetes in geriatric patients are also not entirely evidence-based; instead, they are often based on the clinical experiences of the expert panel involved in developing the guidelines [17]. A recent study comparing insulin regimens of geriatric and non-geriatric patients in actual clinical practice reported a discrepancy between practice and guideline recommendations [18]. The authors of this study noted that geriatric subjects were more commonly treated with simple regimens involving greater use of basal insulin instead of fast-acting insulin. The time-action profiles of rapid-acting insulin have been shown to better mimic the physiological response of endogenous insulin to food intake compared with regular human insulin [18–20]. As a result, some reports suggest that the rapid-acting insulin analogs may be well-suited for optimal glycemic control in geriatric populations [16, 20–22]. Our current findings support the contention that the rapid-acting insulin analog insulin lispro is as effective in achieving glycemic control in geriatric patients as it is in non-geriatric patients with type 2 diabetes at a similar dose.

Due to the potential for adverse effects, safety considerations with glycemic control are always crucial in the management of diabetes. Hypoglycemia is the most frequent undesirable effect of insulin therapy. Obtaining optimal glycemic control while preventing hypoglycemia is a particular challenge clinicians face when treating geriatric patients, partly due to the difficulty in predicting the timing of peak insulin action [21]. Because frail geriatric patients (distinguished from autonomous patients free of serious co-morbidity using evaluation scales validated for geriatric patients) are particularly at increased risk for hypoglycemia, especially with aggressive therapeutic goals, many guidelines (as reviewed by Constans [17]) recommend distinguishing frail patients from patients free of comorbidity prior to establishing treatment goals. The fear of hypoglycemia also tends to make some patients accept suboptimal glycemic control. While our current analysis did not distinguish between frail versus non-frail patients within the geriatric population, our analysis demonstrates that insulin lispro was effective in improving glycemic control in geriatric patients with similar incidence and rate of hypoglycemia as compared to non-geriatric patients.

Also, the incidence of severe hypoglycemia is reported to be higher in geriatric patients compared with younger patients when treated with insulin alone [18]. Since no cases of severe hypoglycemia were reported in one of the two studies containing the definition [13], our analysis of severe hypoglycemia was limited to data from just one study that included only patients with a documented history of acute myocardial infarction [12]. Although the overall incidence of severe hypoglycemia was low in the current analysis, a statistically significant greater proportion of patients in the geriatric cohort had at least one episode of severe hypoglycemia compared with non-geriatric patients. As reported in the literature, it is not an uncommon observation to find an increased frequency of severe hypoglycemia in geriatric patients [23–29]. Indeed, in a study analyzing data from two recent cross-sectional surveys, nearly 10 % of the study population was reported to have had an event of severe hypoglycemia at least once a year [28], and more frequently in patients with considerable comorbidity undergoing aggressive diabetes management [25]. Although flexibility of glycemic goals is being contemplated in the recently updated ADA guidelines (2012), and has been considered as a reasonable approach in trying to reduce the overall risk of hypoglycemia in geriatric populations [30], the study reporting severe hypoglycemia [12] was conducted with a goal of achieving and maintaining HbA_1c_ <7 % as per the ADA guidelines at that time.

As shown in the literature, a great number of hypoglycemic episodes (including severe hypoglycemia) may be avoided by educating patients on the principles of blood glucose monitoring, by involving general practitioners in outpatient management of diabetes, and by close monitoring of hypoglycemia [25, 26, 31]. Our current meta-analysis demonstrates that geriatric patients can be treated safely with insulin lispro, with no age-related differences in either the rate or the incidence of overall hypoglycemia.

Our current study has some limitations. The primary limitation is that the data analysis was retrospective and included only patients with type 2 diabetes. Therefore, the results may not be generalizable to the entire geriatric patient population with diabetes, including type 1 diabetes. Also, the studies included in the meta-analysis were relatively short-term and had sample sizes too small to draw conclusions relative to cardiovascular events. Analyses of hypoglycemic episodes were based on the respective definitions of hypoglycemic episodes used in the individual studies, which were inconsistent across the seven studies analyzed. This inconsistency may have resulted in hypoglycemic episodes being captured in some studies, but not in others, thus making it difficult to compare hypoglycemia across studies. In addition, the interaction between kidney function and hypoglycemia could not be assessed based on the GFRs and plasma creatinine levels. Instead, the incidences of hypoglycemia were reported after adjusting renal function status.

In conclusion, the results of this retrospective meta-analysis suggest that geriatric patients with type 2 diabetes can be treated with insulin lispro to achieve the same level of metabolic control as in non-geriatric patients. The general safety profile relating to hypoglycemia was similar in the two age cohorts; thus, suggesting that insulin lispro is a safe and effective treatment option for the geriatric population.
